# One‐Pot Synthesis of Melt‐Processable Supramolecular Soft Actuators

**DOI:** 10.1002/anie.202115166

**Published:** 2021-12-20

**Authors:** Sean J. D. Lugger, Dirk J. Mulder, Albertus P. H. J. Schenning

**Affiliations:** ^1^ Stimuli-responsive Functional Materials and Devices Department of Chemical Engineering and Chemistry Eindhoven University of Technology P.O. Box 513 5600 MB Eindhoven The Netherlands; ^2^ Institute for Complex Molecular Systems Eindhoven University of Technology Den Dolech 2 5600 MB Eindhoven The Netherlands

**Keywords:** hydrogen bonds, liquid crystals, segmented copolymers, soft actuators, supramolecular polymers

## Abstract

The application of reprocessable and reprogrammable soft actuators is limited by the synthetic strategies, 3D‐shaping capabilities, and small deformations. In this work, melt‐processable supramolecular soft actuators based on segmented copolymers containing thiourethane and liquid crystal segments have been prepared via sequential thiol addition reactions in a one‐pot approach using commercially available building blocks. The actuators demonstrated immediate, reversible response and weightlifting capabilities with large deformations up to 32 %. Through exploiting the supramolecular cross‐links, the material could be recycled and reprogrammed into 3D actuators and welded into an actuator assembly with different deformation modes. Our work offers a one‐pot synthesis and straightforward melt‐processable approach to prepare supramolecular soft actuators with large deformations that can be reprocessed and reprogrammed into arbitrary 3D shapes.

## Graphical abstract

A new class of polythiourethane soft actuators based on liquid crystal and thiourethane segments has been developed by a one‐pot approach. The formation of well‐defined thiourethane segments affords supramolecular cross‐links through hydrogen‐bonding interactions yielding materials that can be reprocessed and reprogrammed into arbitrary 3D shapes. A detailed understanding and characterization of the developed material are presented.

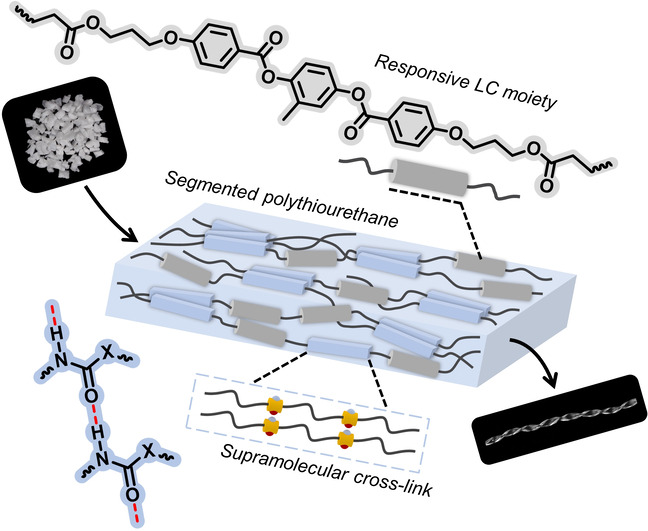

## Introduction

Stimuli‐responsive liquid crystal elastomers (LCEs) are capable of performing fast and reversible actuation, and have readily been applied as soft actuators[^1^, ^2^, ^3^, ^4^, ^5^] in applications such as soft robotics,[^6^, ^7^] smart textiles,[^8^, ^9^] microfluidics,^[10]^ and artificial muscles.[^11^, ^12^] A macroscopic mechanical response arises from an ordered to less ordered state in the covalently cross‐linked network thermosets at the isotropization temperature (*T*_i_). Hence, subjecting the responsive LCEs to an external stimulus such as heat results in a contraction along the oriented mesogens‐based network's director field and expansion perpendicular to it, inducing macroscopic shape changes. After removing the stimulus, the initial molecular order is recovered, and the shape is restored due to the cross‐linked network. A proven method to prepare aligned LCEs is by first mechanically stretching a partially cross‐linked material to induce alignment, followed by fully photo‐crosslinking the polymer locking in the desired molecular orientation of the mesogens.[^13^, ^14^, ^15^, ^16^] Although the currently available materials exhibit large deformations up to 400 % contraction,[^17^, ^18^] have good mechanical properties,^[19]^ and are sufficiently stable,^[20]^ they cannot be reprogrammed and recycled.

One strategy to overcome the limitations inherent to a permanent cross‐linked polymer network is to use dynamic covalent bonds instead. Dynamic covalent LCE networks have been reported demonstrating more versatile processability of the material.[^21^, ^22^, ^23^, ^24^, ^25^] Rearranging the molecular structure of these covalently exchangeable networks is facilitated by a chemical reaction often requiring a catalyst.[^26^, ^27^] While LCE actuators based on dynamic covalent networks are capable of welding, recycling, and reprogramming, materials that are melt‐processable with programmable molecular orientation render new possibilities to enable conventional processing methods using the polymer melt. Only very recently, the dynamic thermal rearrangement of a covalent adaptable network was reported enabling depolymerization and repolymerization, allowing for processing the material in the viscous melt.^[28]^

An alternative strategy to circumvent permanently cross‐linked networks is by introducing supramolecular interactions as dynamic physical cross‐links. Among the potential supramolecular interactions, hydrogen bonds have emerged as one of the most attractive motives, and hydrogen‐bonded liquid crystal (LC) polymers capable of reversible actuation have become an emerging research area.[^29^, ^30^, ^31^, ^32^] However, supramolecular cross‐linked LCE actuators have generally been prepared by multistep synthesis, and reprogramming arbitrary initial shapes has not been reported (Table S1).[^33^, ^34^, ^35^, ^36^] Furthermore, the reversible deformation in these supramolecular LC actuators is limited (<2 % contraction) as the *T*_i_ is above the melting temperature (*T*_m_). Therefore, the actuation strains are low since the *T*_i_ is not passed (vide supra).

**Figure 1:**
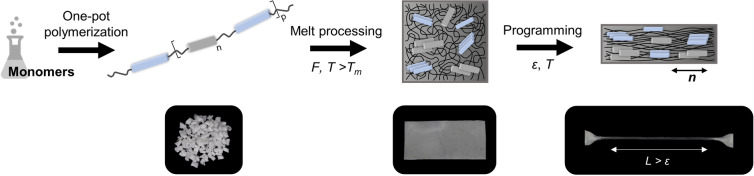
Preparation of supramolecular cross‐linked soft actuators based on thermoplastic PTU as melt‐processable material. One‐pot polymerization of the monomers gives a segmented copolymer and compression molding the obtained material yields polydomain films. Subsequent programming by elongating the material at elevated temperatures allows for a uniform director field (***n***).

Here, we present thermoplastic polythiourethane (PTU) actuators based on segmented copolymers containing thiourethane (TU) hard segments and LC soft segments. In our approach, we focus on a one‐pot synthesis using commercially available building blocks, providing a modular design and avoiding the necessity of monomer synthesis. The PTUs contain hydrogen bonds that form a physically cross‐linked network in a broad temperature range, ensuring sufficient mechanical integrity and excellent mechanical properties, whereas at high temperatures, the thermoplastic behavior of the material is regained, allowing for the preparation of actuators through melt‐processing and thermal programming (Figure 1). Thermoplastic PTUs having different lengths of the soft LC oligomer segments have been synthesized in one step, from which we evaluated the effect of the segmented copolymer composition on structure, mechanical properties, and actuation performance. Since the synthesized copolymers are well microphase‐separated, the *T*_i_ is located well below the *T*_m_, allowing for large reversible deformations up to 32 % contraction. Additionally, the thermoplastic LCE can be reprocessed, reconfigured, and welded into more complex 3D actuators, showing different shape changes.

## Results and Discussion

**Figure 2:**
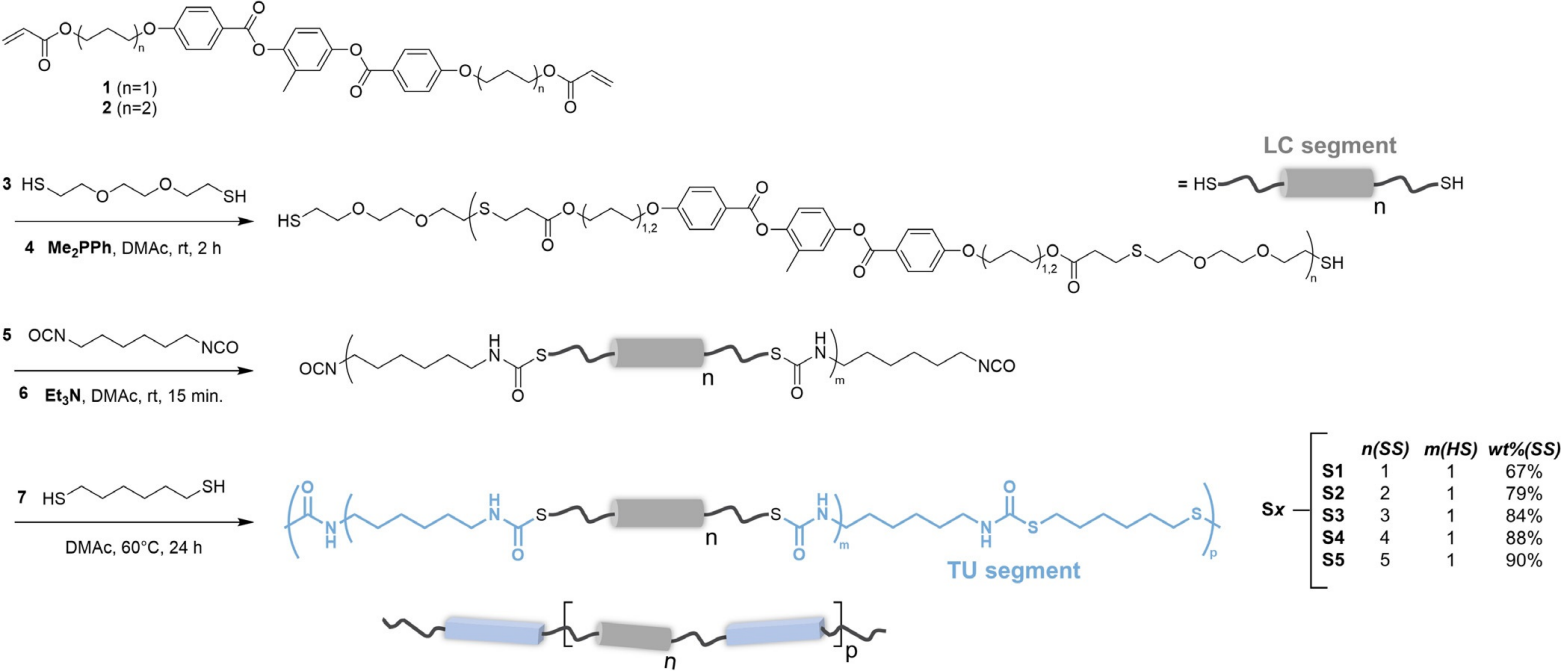
The synthesis of segmented PTUs **S1**–**S5** using sequential addition reactions and the corresponding composition, **S*x***. The blue rectangles and gray rods represent the TU and LC segments, respectively.

To create supramolecular cross‐linked thermoplastic LCEs,^[37]^ main‐chain LC polymers were prepared using sequential thiol‐acrylate and thiol‐isocyanate addition reactions. Commonly used diacrylate mesogens (**1** and **2**) for LCEs, dithiols, and diisocyanate were used as building blocks to prepare the LC TU polymers (Figure 2). A mixture of two mesogens was used to prevent the formation of a smectic mesophase and crystallinity in the LC segment.^[17]^ Inspired by Hoyle and co‐workers, a one‐pot method was used to synthesize segmented PTU materials in which the LC soft segment length was systematically changed.^[38]^ First, the thiol functionalized LC oligomer soft segment was synthesized by a nucleophilic thiol‐Michael addition reaction between an equimolar mixture of diacrylate mesogens (**1** and **2**) and an excess of dithiol (**3**) in the presence of a phosphine catalyst (**4**). Subsequently, in the same flask, a prepolymer was prepared by a base‐catalyzed thiol‐isocyanate addition reaction between the synthesized thiol‐terminated oligomers and an aliphatic diisocyanate (**5**) mediated by an amine catalyst (**6**). In the final stage, the prepolymer was reacted with a difunctional thiol (**7**) to yield linear LC TU polymers. In our experiments, the nomenclature of the PTUs, **S*x***, denotes the calculated mean length of the soft LC segment (**S*x***, i.e., number of mesogens). The mean length of the LC segment chains was controlled from one to five repeating (**S1**–**S5**) units by the stoichiometric ratio of dithiols and reactive mesogens as obtained by theoretical calculations using Carothers’ equation (Table S2). In contrast, the hard TU segment length was maintained constant (*M*_n,theo_=487 g mol^−1^). Moreover, as the soft segment's length and content increase from one to five mesogens per segment, the TU segment content decreases from 33 to 10 wt %, respectively (Table S3). A series of polymers with near‐quantitative yields was obtained by precipitation of the reaction mixture into cold diethyl ether. It should be noted that two different dithiols (**3** and **7**) have been used for the formation of thermoplastic PTUs. The incorporation of only one dithiol (i.e., **3**) in both segments drastically decreased the degree of microphase separation resulting in poor mechanical properties and irreversible shape changes. For all synthesized materials, the formation of polymers was confirmed by Fourier‐transform infrared spectroscopy (FTIR), as indicated by the disappearance of thiol and isocyanate stretching bands at 2560 and 2270 cm^−1^, respectively. Additionally, characteristic amine and carbonyl vibrations were observed for all materials that are indicative of the successful formation of TU moieties. The observed number‐average molecular weight (85 000–157 000 g mol^−1^) and relatively low polydispersity (2.0–2.7) of all materials from gel permeation chromatography (GPC) indicate the successful synthesis of the PTUs (Table S4).

**Figure 3:**
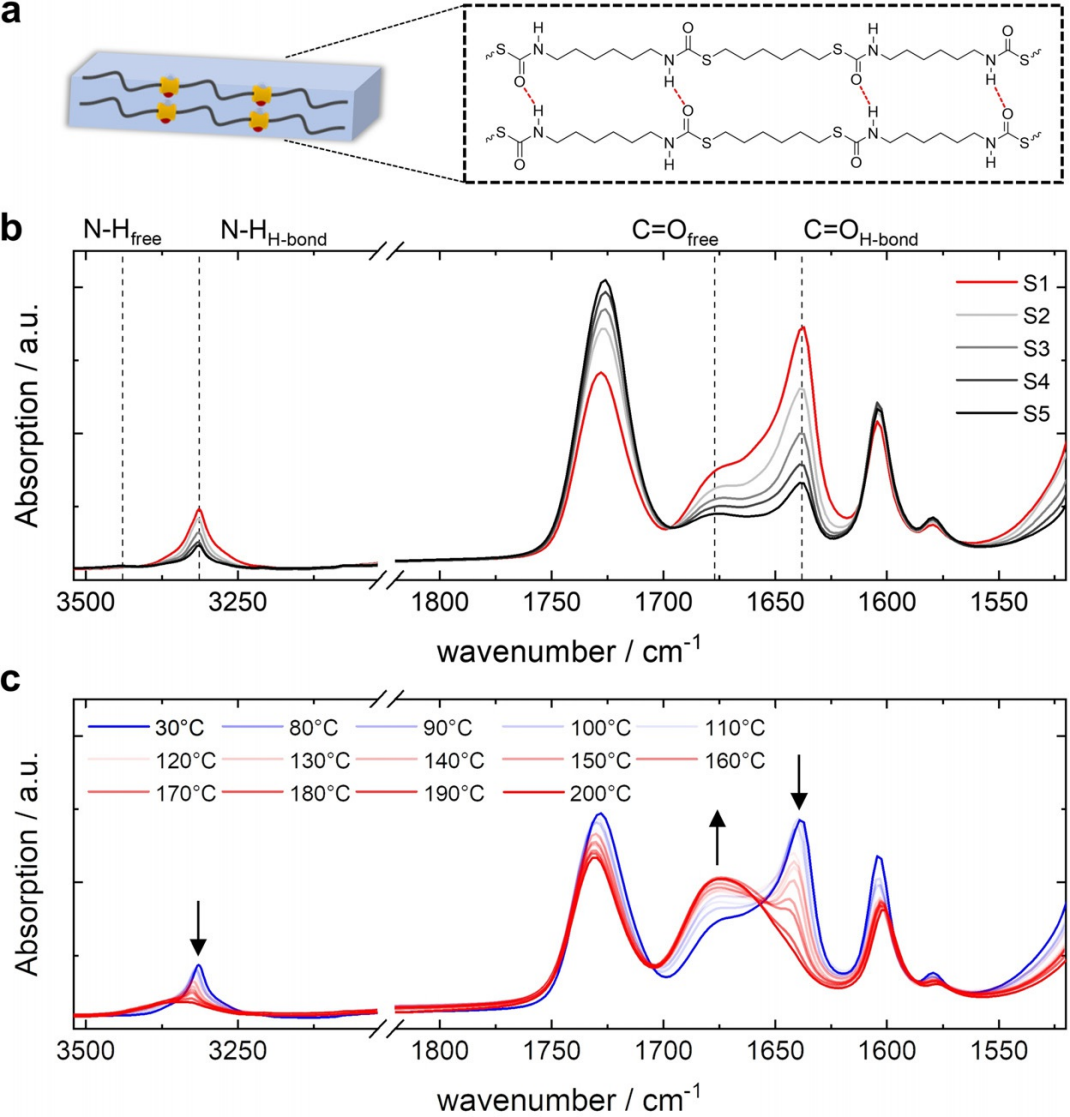
a) Molecular representation of hydrogen‐bonded TU segments in the segmented PTUs. b) FTIR spectra of the PTUs **S1**–**S5** showing free and hydrogen‐bonded TU amine and carbonyl stretching band regions indicated by the dashed lines. c) Temperature‐dependent FTIR spectra of compression‐molded PTU **S1**. The black arrows indicate the increase and decrease of the vibrations upon heating.

The hydrogen bonding properties of the PTUs were investigated by FTIR spectroscopy (Figure 3; Figure S1). The extent of hydrogen bonding within the TU segment was assigned to the absorption in the TU amine (3200–3400 cm^−1^) and carbonyl (1600–1800 cm^−1^) regions (Figure 3 b).[^39^, ^40^, ^41^] Hydrogen‐bonded (N−H_H‐bond_) and free (N−H_free_) TU amine stretching bands were observed at 3315 and 3440 cm^−1^, respectively.^[42]^ The N−H_H‐bond_ stretching band appeared as sharp vibration for all PTUs, while the N−H_free_ could be observed weakly, indicating the formation of well‐organized hydrogen‐bonded TU segments. The ordered hydrogen‐bonded (C=O_H‐bond_) and free (C=O_free_) carbonyl stretching bands of the TU segments were observed at 1638 and 1677 cm^−1^, respectively.[^43^, ^44^] As expected, the absorption of the hydrogen‐bonded TU amine and carbonyl stretching vibrations increased consistently with increasing TU segment content from PTUs **S5** to **S1**. In contrast, the absorption of free TU amine decreased with increasing TU segment content. The absorption band of hydrogen‐bonded carbonyl groups is larger than free carbonyl groups, and the corresponding ratio (C=O_H‐bond_/C=O_free_) increases with the TU segment content (Figure S2). These results suggest that the extent of hydrogen bonding increases at higher TU segment concentrations and indicate a more substantial degree of microphase separation between the LC and TU segments for shorter LC segment lengths. That said, the prepared materials exhibit strong hydrogen bonding interactions that act as supramolecular, physical cross‐links within the TU segment.[^45^, ^46^, ^47^]

When heating the material, the hydrogen bonds remain nearly unaffected up to 110 °C, as indicated by the minor changes in the hydrogen‐bonded and free stretching bands (Figure 3 c). However, at 120 °C, the spectrum exhibits a sudden decrease in absorbance of the hydrogen‐bonded amine and carbonyl stretching vibrations associated with the dissociation onset of the hydrogen bonds. Increasing the temperature further, the hydrogen‐bonded carbonyl peak gradually decreases and shifts to higher wavenumbers until it fully disappears at 200 °C, accompanied by a gradual increase of the free carbonyl peak. The hydrogen‐bonded amine vibration also decreased and shifted to higher wavenumbers. These findings apply to all PTUs and reveal three characteristic temperatures. At temperatures below 120 °C, the hydrogen bonds are maintained, forming a physically cross‐linked network ensuring mechanical stability of the material to preserve its shape and alignment upon thermal actuation. Contrarily, between 120 °C and 200 °C, the hydrogen bonds are partly broken, allowing for strain‐induced alignment of the PTUs that is fixed upon cooling owing to the thermo‐reversibility of the hydrogen bonds. Furthermore, the physical cross‐links are almost entirely absent at 200 °C, ensuring melt‐processable capabilities (vide infra).

The thermal properties of the developed materials were assessed using differential scanning calorimetry (DSC) and thermogravimetric analysis (TGA). The DSC thermograms showed a transition ranging from −15 to −6 °C during heating, assigned to the glass transition temperature (*T*_g_) of the LC segments (Table S5 and Figure S3). The *T*_g_ shifts to lower temperatures when increasing the LC segment chain length from **S1** to **S5** due to decreased physical cross‐link density and an increase in segmental mobility of the LC segment. Endothermic melting peaks (*T*_m_) of the TU segment domains were observed at 164 to 177 °C. Increasing the TU segment content from **S5** to **S1**, the area under the *T*_m_s, and therefore the enthalpy of the transitions, increases as well. These results showed the existence of distinct LC and TU segments in the PTUs. Furthermore, an exothermic peak is observed for PTUs **S3** to **S5** around 35 °C, independent of the LC segment content. The presence of liquid crystallinity was determined with polarized optical microscopy (POM), in which the birefringence disappeared over a broad temperature range (60 to 120 °C) upon heating (Figure S4). The material became birefringent again when cooled, showing thermal hysteresis at the phase transition. These observations reveal that the *T*_m_ of the segmented thermoplastic LCE is higher than the *T*_i_, allowing for optimal actuation performance (vide infra). However, the *T*_i_ is not observed with DSC probably because the transition from the LC ordered to disordered state is broad. The TGA profiles showed two transitions for all materials corresponding to the degradation of TU and ester moieties (Figure S5).^[43]^ The thermoplastic PTUs exhibit a 1 % weight loss around 245 °C due to the decomposition of the polymer, which is well above the processing temperatures indicating the desired thermal stability.

**Figure 4:**
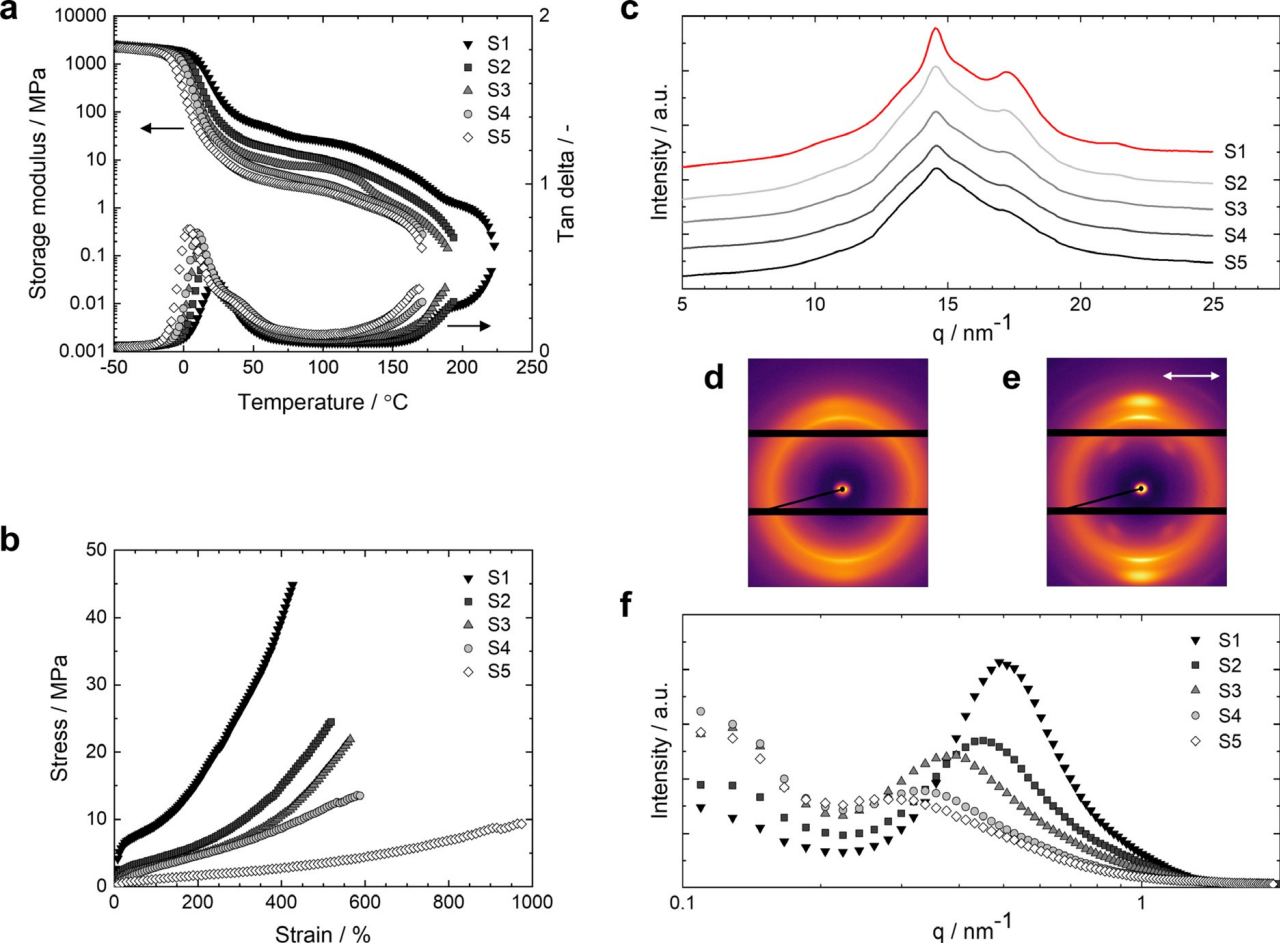
a) DMA storage modulus (*E*′) and loss tangent (tan *δ*) profiles as a function of temperature of the compression‐molded PTUs **S1**–**S5**. The black arrows denote the corresponding axis. b) Stress–strain curves at room temperature of the compression‐molded PTUs **S1**–**S5**. c) 1D WAXS diffractograms of the aligned PTUs **S1**‐**S5**. 2D WAXS diffractograms of PTU **S1** d) before and e) after programming. The white arrow denotes the alignment direction. f) 1D MAXS diffractograms of the aligned PTUs **S1**–**S5**.

Dynamic mechanical analysis (DMA) was performed on compression‐molded samples (8×5.3×0.4 mm^3^) to characterize the dynamic viscoelastic properties of the thermoplastic PTUs (Table S5). In the thermogram, storage modulus (*E*′) inflection points and loss tangent (tan *δ*) peak maximum were observed around 10 °C, corresponding to α‐relaxation of the LC segment (Figure 4 a).[^48^, ^49^] Moreover, a shoulder was observed at approximately 40 °C for PTUs **S3** to **S5**. The rubbery plateau was observed between 65 to 150 °C. Upon increasing the LC segment length and decreasing the TU segment content from **S1** to **S5**, the α‐relaxation transition shifts to lower temperatures, and the magnitude of *E*′ at the rubbery plateau region decreases consistently. This decrease in temperature and rubbery modulus arises from the decreasing concentration of TU segment domains, which act as physical cross‐links.^[50]^ The onset of the melting transition of crystalline TU segment domains, where the material enters the viscoelastic flow region, is revealed by the temperature at which a distinct increase in tan *δ* and a decrease in *E*′ are observed. When increasing the temperature further, the materials show viscous flow, and the elastic properties are lost. The observed α‐relaxation and melting temperature transition trends are consistent with the obtained *T*_g_ of the LC segment and *T*_m_ of the TU segment in DSC, respectively. Similar to the temperature‐dependent FTIR measurements, it can be seen from the DMA temperature profiles of tan *δ* that the developed materials exhibit three distinct temperature regions. At the rubbery region, the tan *δ* is constant, indicating that the physical cross‐links are maintained and the material acts as a covalently cross‐linked system. In the viscoelastic flow region, the supramolecular network is partially broken allowing for strain‐induced orientation of the material upon stretching, whereas at higher temperatures it becomes melt‐processable due to the viscous flow.

Tensile tests were performed on compression‐molded samples (20×2×0.4 mm^3^) to study further the mechanical properties and the LC segment length effect (Table S6). From the stress–strain curve, it was observed that the Young's modulus decreased with increasing LC segment size from **S1** to **S5** (Figure 4 b), which is in line with the observed trend of the rubbery plateau in DMA. Moreover, the plateau region in the stress–strain curve becomes gradually longer at larger LC segment lengths. The elongation at break increases and the tensile strength decreases accordingly. These observations arise from the lower concentration of physical crosslinking points in the material due to the relative decrease of TU segment content.^[51]^ The observed plateau in the stress–strain curve is characteristic for LCEs at which the polydomain material is gradually oriented along the stretching direction, essential for the preparation of aligned LCEs with programmed alignment.^[52]^

In order to demonstrate the reversible shape morphing behavior of the developed thermoplastic PTU LCEs, aligned actuators were prepared first (Figure 1). Polydomain samples were obtained by compression molding the materials at 200 °C, which is well above the melt transition and below the degradation temperature. The compression‐molded polydomain samples were uniaxially elongated (*ϵ*=100 %) at room temperature and subsequently annealed at 130 °C for 30 minutes, at which the physical cross‐links are highly dynamic as this temperature is just above the onset of hydrogen‐bond dissociation. Since the material is programmed at higher temperatures (*T*>*T*_i_), where the molecular order is reduced, the stretched thermoplastic PTUs elongate when cooled to room temperature due to spontaneous organization of the LC segment into the ordered mesophase. After this process, the resulting oriented PTUs appear transparent (Figure 1). In POM, the stretched material exhibited birefringence upon rotating the sample 45° between cross polarizers, indicating alignment (Figure S6).

To verify the molecular orientation and segmental organization, the aligned PTUs were studied with wide‐ and medium‐angle X‐ray scattering experiments (WAXS and MAXS). It is important to note that the oriented samples were measured without an externally applied load. In the wide‐angle diffractograms, multiple diffraction peaks at *q*=10.2, 14.5, 17.2, and 21.3 nm^−1^ and an amorphous halo were observed due to the scattering of LC segment chains and TU segment moieties (Figure 4 c). The diffraction became weaker upon decreasing the TU segment content from 33 wt % (**S1**) to 10 wt % (**S5**). Therefore, it is conceivable that these peaks correspond to the scattering of TU moieties suggesting the formation of well‐crystallized TU domains and increased crystallinity of the PTUs at higher TU segment content. The diffraction peaks at *q*=14.5 and 17.2 nm^−1^ correspond to *d*‐spacings of 0.43 and 0.37 nm, respectively, which are assignable to the characteristic intermolecular spacings between the TU moieties in the hydrogen‐bonding direction and orthogonally. Both the mesomorphic soft segment chains and TU hard segment moieties undergo strain‐induced orientation upon stretching during the orientation process, as indicated by the stronger diffraction peaks in the diffraction profiles of aligned PTUs (Figure 4 c), compared to unaligned PTUs (Figure S7). The 2D WAXS diffractograms of the initial polydomain samples showed ring‐shaped, isotropic patterns suggesting the random orientation of the segmented domains (Figure 4 d; Figure S8). In contrast, the wide‐angle 2D X‐ray diffraction patterns of the oriented PTUs showed typical orientationally arranged diffraction spots orthogonal to the elongation direction (Figure 4 e; Figure S9). These patterns arise from the associated scattering of well‐aligned LC and TU domains facilitated by the director alignment of oriented PTUs. The aligned samples also exhibit a four‐point pattern at an azimuthal angle of 60° from the stretching direction (Figure S10), attributed to the scattering of TU structures. It is believed that the TU moieties adopt a chevron‐like geometry that is oriented along the orientation direction upon stretching, resulting in four‐point scattering. The corresponding scattering peak was observed at *q*=10.2 nm^−1^ (*d=*0.62 nm) and reflected the characteristic length of TU moieties. The above observations show that the material is aligned during the orientation process yielding anisotropic PTUs. Moreover, the aligned PTUs show an order parameter between 0.39 and 0.43 with the same degree of stretching during the orientation process (Figure S11). In contrast, unaligned samples exhibit a diffuse halo with a very low orientational order, likely induced by sample handling. It should be noted that the order parameter is measured of the whole material since the observed signals of the LC and TU segments were overlapping. In the small‐angle region of the medium‐angle diffractogram, a peak originating from the interdomain spacing is observed due to the formation of microphase‐separated morphologies (Figure 4 f). With increasing LC segment length, the interdomain spacing of the microphase‐separated segments increases for elastomers **S1** and **S5** from 12.6 to 22.4 nm, respectively. On the other hand, the intensity of the signal is reduced as the relative amount of TU segment becomes less with increasing LC segment length. Comparing the MAXS diffraction profiles before and after aligning the PTUs shows a similar interdomain spacing trend indicating that the microphase‐separated morphology is unaffected upon forming oriented PTUs (Figure S12). The apparent interdomain spacings indicate the formation of well‐defined microphase‐separated structures consisting of LC and hydrogen‐bonding TU domains that act as reversible switching segments and supramolecular cross‐links in the thermoplastic PTU, respectively.

**Figure 5:**
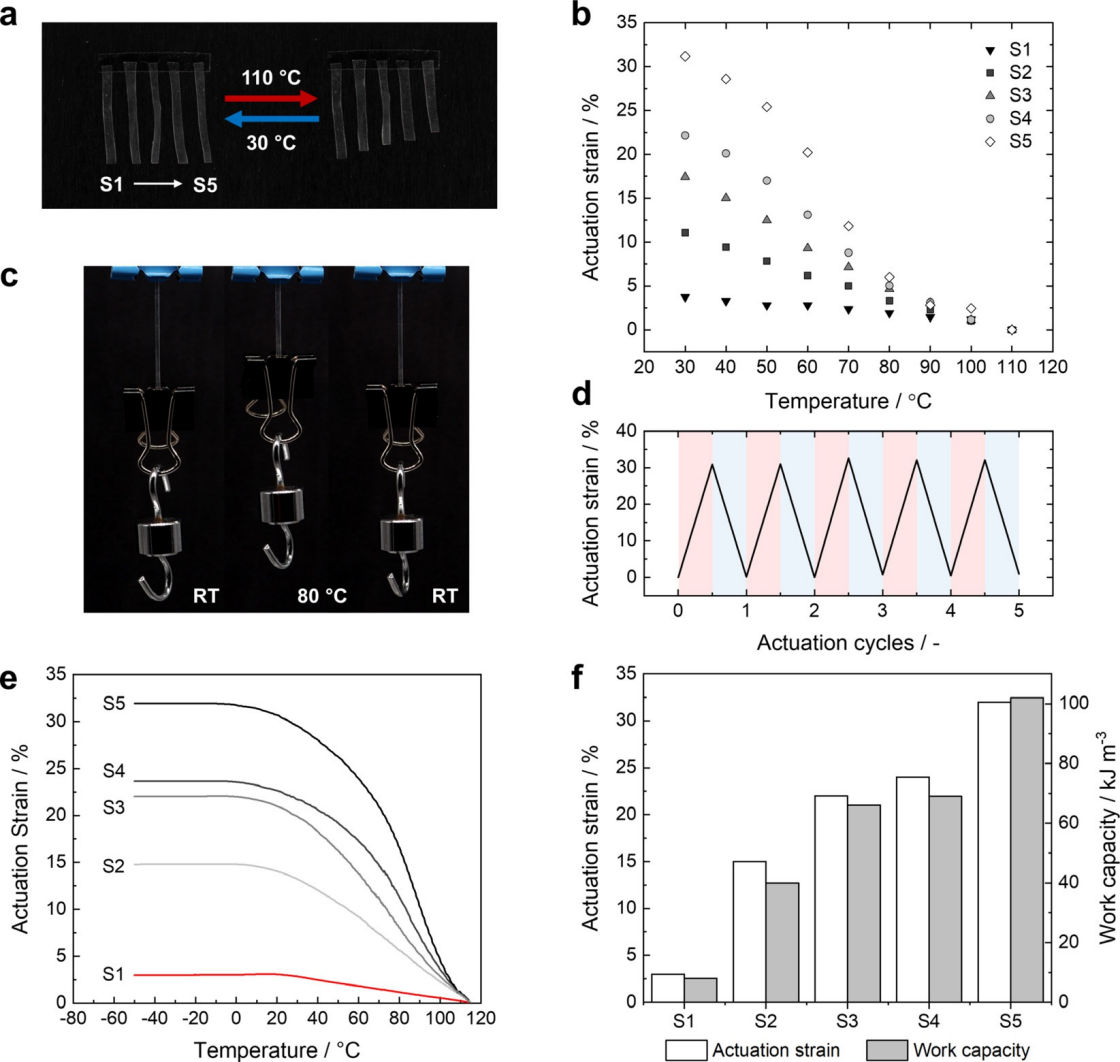
a) Images of the PTU actuators **S1–S5** (from left to right) at 30 °C and 110 °C. The actuators are fixed to the surface at the top. b) The corresponding actuation strain as a function of temperature. c) Images of PTU **S5** lifting an additional weight (5 g) when heated to 80 °C. d) Actuation strain of PTU **S5** with a load (5 g) upon cycling between room temperature and 80 °C. The red and blue boxes correspond to the heating and cooling of the actuator, respectively. e) Thermal actuation of the aligned PTUs **S1–S5** under constant bias stress (250 kPa). f) Maximum actuation strain ratio and corresponding actuation work capacity for each PTU.

Unbiased reversible shape changes of the oriented thermoplastic LCEs were observed when heated and cooled between 30 °C and 110 °C showing a maximum actuation strain ratio of 32 % (Figure 5 a,b). The deformation mechanism in these supramolecular materials is identical to classic LCEs; temperature‐induced disorder of the LC moieties results in contraction along the director field and perpendicular expansion (vide supra). Upon heating above 130 °C, the strain‐induced orientation is erased, and the reversible actuation behavior is lost. Therefore, the PTU actuators are heated to a maximum temperature of 110 °C, at which the extent of hydrogen bonding is maintained (vide supra). In the thermal cycling experiments, the actuator instantly contracted along with the programmed director field when heated and completely recovered to the initial length upon cooling. The magnitude of actuation strain increases significantly when increasing the LC segment length from PTU **S1** to **S5** due to the higher LC content and decreasing stiffness of the material. This indicates that the LC segment content dominates the thermal actuation behavior. To demonstrate the developed actuators’ immediate reversible response and weightlifting capabilities, PTU **S5** was repeatedly heated and cooled while loaded with various weights. The loaded actuator rapidly contracted when heated to approximately 80 °C and completely recovered to its initial length upon removing the heat source (Figure 5 c). Moreover, the thermoplastic LCE actuator loaded with 5 grams of weight exhibited fully reversible actuation over at least five heating and cooling actuation cycles, indicating the maintained orientation and thermal stability of the supramolecular cross‐linked actuator (Figure 5 d; Video S1). As loading is increased from 5 to 30 grams, the actuation strain gradually decreases until, eventually, the sample hardly contracts (Video S2). The thermal actuation performance of oriented thermoplastic LCEs was further characterized when subjected to an initial bias stress of 250 kPa as a function of temperature in the DMA (Figure 5 e). The observed actuation exhibits a similar trend and magnitude of actuation strain for all samples observed for unbiased actuation before. Additionally, the actuator's work capacity was calculated with a maximum energy density of 102 kJ m^−3^_,_ corresponding to an actuation strain of 32 % for PTU **S5** (Figure 5 f).

**Figure 6:**
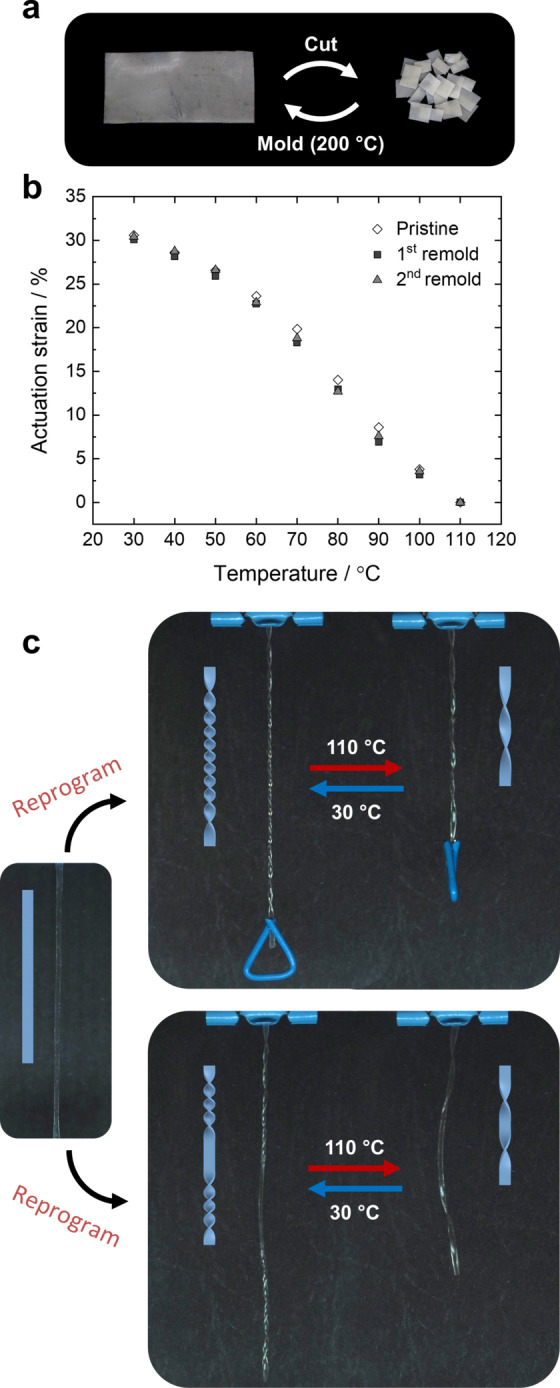
a) Reprocessing cycle of PTU **S5** illustrating cutting and remolding of the film. b) Actuation strain as a function of temperature of pristine and remolded samples. c) Reprogramming of aligned **S5** into arbitrary 3D geometries, i.e., a left‐handed twisted actuator (top panel) and a patterned actuator with a left‐ and right‐handed twisted segment divided by a uniaxial programmed region (bottom panel) and the corresponding temperature response.

Finally, utilizing the hydrogen‐bonding motives’ dynamic character, the PTU material can be recycled, reprogrammed, and welded, illustrating the advantage of using physical cross‐links. To demonstrate the recyclability, a pristine film of PTU **S5** was cut into small pieces and remolded up to two times at 200 °C (Figure 6 a). The reprocessed material exhibits similar mechanical properties with an increase in elongation at break and tensile strength as observed from the stress–strain curves (Figure S13). Upon heating and cooling, the pristine and recycled materials show the same actuation behavior (Figure 6 b). By reprogramming the shape of an aligned actuator, the material can be reconfigured and actuated again, showing different types of deformation. A twisted actuator[^53^, ^54^] was prepared by thermally reprogramming a uniaxially aligned material in a twisted configuration at 130 °C for 30 minutes (Figure 6 c). Upon cooling to room temperature, a twisted actuator having 16 left‐handed twists is obtained. The twisted actuator contracts and untwists to 6 turns upon increasing the temperature while lifting a load (0.127 g; Video S3). During cooling, the actuator regains its shape and molecular alignment into its initial conformation. The 3D twisted shape of the reprogrammed actuator is a result of the hydrogen‐bonds dynamic character, allowing for the exchange of supramolecular cross‐links at increased temperatures that are fixed upon cooling in the predetermined shape. The obtained actuator likely employs a tilted molecular alignment of the LC moieties upon reprogramming, allowing for contraction and untwisting deformations due to temperature‐induced disorder of the LCs by heating. Similarly, a more complicated 3D shape was prepared to consist of a left‐ and right‐handed twisted segment divided by a uniaxial programmed region (Figure 6 c). This allows for a more exotic thermal deformation in which all programmed regions of the actuator reversibly contract and expand, while the predetermined twists also show reversible untwisting and twisting motions upon heating and cooling (Video S4). Additionally, it is shown that two programmed shapes can be welded together to form one single actuator. A uniaxially and a twisted programmed actuator are welded together perpendicularly into a different geometry by locally overlapping and heating the ends to 200 °C for 2 minutes. The welded actuator shows reversible temperature‐triggered actuation and different deformation modes for each individual part being untwisting and/or contraction (Video S5). Accompanying the untwisting motion, the welded actuator bends upon heating since it is fixed on one side to the surface. The ease of reprogramming and welding allows for the fabrication of more complex 3D shape designs and accompanying deformations.

## Conclusion

We have successfully developed a new generation of melt‐processable supramolecular cross‐linked LCEs based on segmented PTU and demonstrated a processing method to obtain actuators by molding and stretching. The formation of well‐defined TU domains affords supramolecular cross‐links in the material through hydrogen bonding, providing the desired mechanical stability during actuation, whereas the reversibility of the hydrogen bonds allows for melt‐processable materials with programmable molecular alignment. This approach is in line with typical processing methods for thermoplastic polymers using the polymer melt and allows for aligning the PTUs by enabling the processability of the network upon heating. Due to the segmented structure, the thermoplastic LCE is responsive below its melting temperature (*T*_i_<*T*_m_), allowing for optimal actuation performance. When heated, the PTU actuators undergo reversible contraction capable of lifting a load. Reversible contractions up to 32 % were achieved, of which the deformation depends on LC segment length. Furthermore, reprocessing of a PTU film has been demonstrated, and reconfiguration of an actuator into another arbitrary 3D geometry exhibiting a different shape change upon applying an external stimulus is achieved through reprogramming and welding. We anticipate that these supramolecular cross‐linked LCEs offer an innovative approach towards (re)programmable and recyclable stimuli‐responsive materials for applications ranging from smart surfaces to soft robotic devices. The properties of these soft actuators can be easily further tuned and expanded by the currently available reactive LC chemical toolbox and building blocks typically used for thermoplastic elastomers.

## Conflict of interest

The authors declare no conflict of interest.

## Supporting information

As a service to our authors and readers, this journal provides supporting information supplied by the authors. Such materials are peer reviewed and may be re‐organized for online delivery, but are not copy‐edited or typeset. Technical support issues arising from supporting information (other than missing files) should be addressed to the authors.

Supporting Information

Supporting Information

Supporting Information

Supporting Information

Supporting Information

Supporting Information
