# Supplementary material for: One‐Pot Synthesis of Melt‐Processable Supramolecular Soft Actuators
Source: Angew Chem Int Ed Engl. 2021 Dec 20;61(6):e202115166. doi: 10.1002/anie.202115166 (PMC9300041; doi:10.1002/anie.202115166)
Supplement: Supplementary file 1 — Supporting Information [file ANIE-61-0-s005.pdf]

Angewandte Chemie
Eine Zeitschrift der Gesellschaft Deutscher Chemiker

Supporting Information

One-Pot Synthesis of Melt-Processable Supramolecular Soft Actuators

Sean J. D. Lugger, Dirk J. Mulder, and Albertus P. H. J. Schenning**

anie_202115166_sm_miscellaneous_information.pdf
anie_202115166_sm_Video_S1.mp4
anie_202115166_sm_Video_S2.mp4
anie_202115166_sm_Video_S3.mp4
anie_202115166_sm_Video_S4.mp4
anie_202115166_sm_Video_S5.mp4

Table of Contents

Experimental Procedures 2
Supplementary Tables and Figures 3
Supplementary Video Captions 10
References 10
Author Contributions 10

Experimental Procedures

Materials
1,4-Bis-[4-(3-acryloyloxypropyloxy)benzoyloxy]-2-methylbenzene (**1**) and 1,4-Bis-[4-(6-acryloyloxyhexyloxy)benzoyloxy]-2-methylbenzene (**2**) were obtained from Merck. Dimethylphenylphosphine (**4**, 99%) and *N,N*-Dimethylacetamide (DMAc, ≥99%) were purchased from Sigma-Aldrich. 2,2′-(Ethylenedioxy)diethanethiol (**3**, ≥97%), Hexamethylene diisocyanate (**5**, ≥98%), Triethylamine (**6**, ≥99%), and 1,6-Hexanedithiol (**7**, ≥97%) were purchased from Tokyo Chemical Industry (TCI). Diethyl ether (Et_2O, ≥99.5%) was obtained from Biosolve. All reagents were used as received without further purification.

Synthetic procedure
A reaction vessel (100 mL) was charged with diacrylate mesogens **1** and **2** in DMAc (50 wt%) and allowed to stir under an inert atmosphere at 50 °C until fully dissolved. The solution was cooled to room temperature, and dithiol chain extender **3** was added while stirring, followed by nucleophilic catalyst **4** (0.1 wt%). The resulting reaction mixture was allowed to react at room temperature for 2 h. Afterward, diisocyanate **5** in DMAc (50 wt%) was added to the oligomer mixture immediately followed by base catalyst **6** (0.1 wt%) and allowed to stir at room temperature for 15 minutes. During this time, the mixture became viscous, and additional DMAc (30 wt%) was added to the prepolymer mixture. Next, dithiol **7** was added dropwise, and after complete addition, the reaction mixture was heated at 60 °C and allowed to react overnight. The crude mixture was poured into cold Et_2O (500 mL) while stirring vigorously, and the polymer precipitated over time. The product was added to fresh Et_2O (200 mL) and stirred overnight. The solvent was decanted, and the final polymer was dried at 40 °C under vacuum affording a white solid (≥97% recovery). The molar ratios and formulations of the synthesized thermoplastic PTU LCEs can be found in the Supporting Information (Table S2, S3).

Characterization
FTIR spectra were recorded on a Varian 670 IR spectrometer equipped with an attenuated total reflectance (ATR) sampling accessory using a diamond crystal over a range of 4000-650 cm^{-1} with 50 scans per spectrum and a spectral resolution of 4 cm^{-1}. All spectra were recorded at room temperature unless stated otherwise. The obtained spectrums are processed with Varian Resolutions. Gel permeation chromatography (GPC) was performed on a Waters HPLC system equipped with a PSS PFG (8 × 50 mm, 7 µm) and two PFG linear XL columns (8 × 300 mm, 7 µm) in series. 1,1,3,3,3-hexafluoro-2-propanol (HFIP) with potassium trifluoroacetate (20 mM) at 35 °C was used as mobile phase supplied at a flow rate of 0.8 mL min^{-1}. The samples were prepared in HFIP with potassium trifluoroacetate (20 mM) and toluene (20 mM) at room temperature. The molecular weights were determined using a refractive index detector relative to poly(methyl methacrylate) standards. DSC measurements were performed using a TA Instruments Q1000 DSC instrument with hermetic T-zero aluminum sample pans. All scans were conducted with 10 ± 1 mg polymer over a temperature range from -50 to 200-210 °C at heating and cooling rates of 10 °C min^{-1} under nitrogen atmosphere. The second heating and cooling cycles were used to determine the enthalpies and transition temperatures of all samples. TGA was carried out on a TA instruments Q50 instrument with 4 ± 0.5 mg polymer over a temperature range from 28 to 800 °C and a heating rate of 5 °C min^{-1}. DMA was performed on samples (8 × 5.3 × 0.4 mm^3) cut from compression-molded films with a TA Instruments Q800 apparatus in vertical tension mode. The thermographs were measured from -50 to 250 °C with a heating rate of 5 °C min^{-1}, a single 1 Hz oscillating frequency, 10 µm amplitude, and 0.01 N preload force. Stress-strain curves were obtained with a Lloyd-Ametek EZ20 tensile testing machine using a 500 N load cell. The strain is defined as $(l-L)/L$ where L is the initial length, and l is the length at a particular time. Dog-bone specimens with a cross-sectional area of 2 × 0.4 mm^2 (W × T) were cut from compression-molded films and uniaxially elongated at an elongation rate of 10 mm min^{-1} with a gauge length of 20 mm until failure. X-ray scattering measurements were performed on a Ganesha lab instrument equipped with a Genix-Cu ultralow divergence source that generates X-ray photons with a wavelength and flux of 0.154 nm and 1 × 10^8 photons s^{-1}, respectively. Diffraction patterns were obtained using a Pilatus 300 K silicon pixel detector with 487 × 619 pixels of 172 × 172 $µm^2$. Silver behenate was used as a calibration standard. The sample-to-detector distance was 89 mm for wide-angle (WAXS) configurations, whereas, for medium-angle (MAXS), the detector was operated at 439.5 mm. The collected data were reduced and analyzed using a custom Python script with the PyFAI software package. D-spacings were calculated using the equation $d = 2\pi/q$. The orientational order parameters were calculated from the diffraction patterns using the Kratky method.[1] POM was carried out with a Leica DM2700 M microscope and crossed polarizers. Isotropization temperatures were determined on drop-casted samples from HFIP (15 ± 1 mg mL^{-1}) using a Linkam THMS600 microscope stage with a heating and cooling rate of 10 °C min^{-1}.

Fabrication procedures
First, the polymer was dried at 60 °C for at least 1 h before processing. Then, the material was loaded homogenously into a mold (20 × 40 × 0.1 mm^3) and covered with polytetrafluoroethylene (PTFE) protection sheets (T = 0.12 mm) on both sides. The mold was heated to 200 °C in a Collin P200E press and subjected to five breath cycles with a mold pressure of 50 bar. The final compression molding process was performed at 100 bar and 200 °C for 2 minutes, whereafter the mold was immediately quenched to room temperature to form polydomain polymer films. Dog-bone shaped specimens (35 × 2 × 0.4 mm^3) were cut from compression-molded polymer films and uniaxially strained at room temperature by using a custom-made stretching instrument until elongation reached 100%. The strained samples were then heated to 130 °C for 30 minutes and subsequently cooled to room temperature while remaining strained. It was noticed that during cooling, the aligned samples spontaneously elongated above the initially applied strain. Finally, the aligned LCEs were annealed at room temperature for at least 48 h before characterization and testing. For recycling, a film was cut into small pieces and compression-molded according to the previously described procedure. The twisted actuator was obtained by fixing one end of an aligned LCE while twisting and subsequently fixing the other followed by heating it to 130 °C for 30 minutes, after which it was cooled to room temperature. Similarly, a uniaxial region was fixed in between a left- and right-handed twisted segment by using two tweezers during programming. Welding was performed by overlapping the end of two actuators and heating it to 200 °C for 2 minutes.

Actuation measurements
Thermal actuation measurements were performed by placing aligned samples on a black anodized aluminum sheet on top of a hotplate. The samples were heated from 30 to 110 °C by gradually increasing the temperature in intervals of 10 °C. Afterward, the samples were allowed to cool to room temperature. All samples were subjected to a full heating and cooling cycle to erase the thermal history before the actuation measurement. Photographs were taken at each temperature using a camera (Olympus OM-D E-M10 Mark III), and the obtained images were analyzed using ImageJ. Weightlifting tests were conducted with various weights attached to the sample with a paper clamp (1.26 g) and heated to around 80 °C using a heat gun. Actuation measurements of reprogrammed actuators were performed in an oven with a window allowing for recording the deformations upon heating and cooling. Actuation strain was measured on a TA Instruments Q800 DMA by monitoring the sample length as a function of temperature. Samples (8 × 1.3 × 0.3 mm^3) were measured in controlled force mode from -50 to 110 °C at a heating rate of 5 °C min^{-1} under initial bias stress of 250 kPa (constant force). All samples were heated to 110 °C for 3 minutes before the measurement to ensure the thermal history was erased. The corresponding work capacity was calculated from Equation 1, considering that the bias stress depends on the cross-sectional area of the samples, which changes upon actuation.[2]

$$\text{Work capacity} = \frac{W}{V} = \frac{F_{\text{bias}}}{WT}\frac{\Delta L}{L} = \sigma_{\text{bias}}\varepsilon_{\text{bias}}\ [kJ\ m^{-3}] \quad (1)$$

Supplementary Tables and Figures

Table S1. Overview of previously reported work describing thermoplastic LC polymer actuators to distinguish and indicate the advantages of this work.

Description	T_m (or T_g)	T_i	Actuation mode	Initial shape	Actuation strain	Ref
Side-chain methacrylate with pending UPy groups	94 – 113 °C [a]	142 – 158 °C	Photo / Bending	Fiber	1.6% [b]	[3]
	N.A.	N.A.	Photo / Bending	Fiber/ film	0.72% [b]	[4]
Azobenzene chain-extended polyurethane doped with azobenzene and gold nanorods	60 – 68 °C	112 °C (doped azo)	Photo / Bending Thermal / Contraction	Film	55% [c]	[5]
Polyurethane with pending LC moieties by amine quaternization	81 °C [a]	135 °C	Photo / Bending	Film	6.6% [b, c]	[6]
Main-chain LC polyurea polymer by thiol-(meth)acrylate addition	N.A.	89 – 105 °C	Photo / Bending	Fiber/ film	0.49% [b]	[7]
Polythiourethane based on sequential thiol-acrylate and isocyanate addition reactions	165 – 177 °C	60 – 120 °C	Thermal / Contraction	3D	32%	This work

[a] Processing is based on the T_g of the material; [b] Calculated from the bending deformations[8]; [c] Irreversible deformation requiring pre-straining after each deformation cycle.

Table S2. Molar ratio of the monomers used for the synthesis of thermoplastic PTUs **S1**-**S5**.

PTU	(1)	(2)	(3)	(5)	(7)
**S1**	1	1	4	2	1
**S2**	1	1	3	2	1
**S3**	1	1	2.67	2	1
**S4**	1	1	2.50	2	1
**S5**	1	1	2.40	2	1

Table S3. Formulation of the thermoplastic PTUs **S1**-**S5**. Number of repeating units (*n*) for LC segment and TU segment length as obtained by theoretical calculation using Carothers' equation. Corresponding theoretical number average molecular weight ($M_{n,theo}$) and content of the LC segment and TU segment.

	LC segment			TU segment		
PTU	*n*	$M_{n,theo}$ [g mol^{-1}]	wt%	*n*	$M_{n,theo}$ [g mol^{-1}]	wt%
**S1**	1	995	67	1	487	33
**S2**	2	1808	79	1	487	21
**S3**	3	2621	84	1	487	16
**S4**	4	3434	88	1	487	12
**S5**	5	4247	90	1	487	10

Table S4. GPC results of the thermoplastic PTUs **S1**-**S5**.

PTU	$M_{n,GPC}$ [g mol^{-1}]	PDI
**S1**	157430	2.66
**S2**	150385	2.41
**S3**	139576	2.28
**S4**	95973	2.18
**S5**	84837	1.96

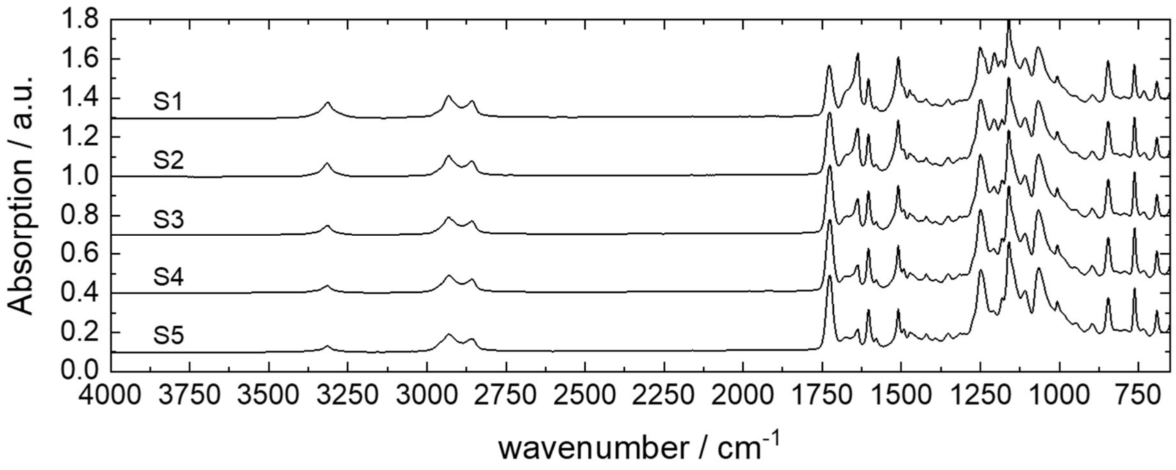

Figure S1. FTIR spectra of polydomain PTUs **S1**-**S5**.

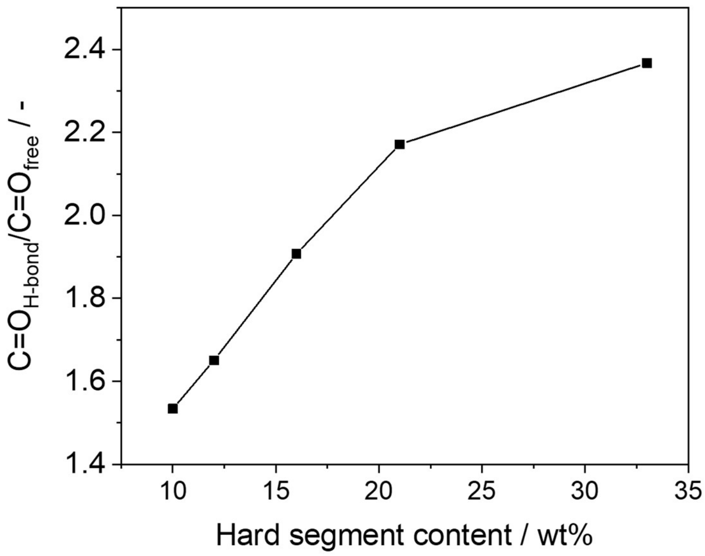

Figure S2. $C{=}O_{H\text{-}bond}/C{=}O_{free}$ ratio as a function of hard segment content.

Table S5. The DSC and DMA results of the thermoplastic PTUs **S1**-**S5**.

	DSC				DMA		
PTU	T_g [°C]	T_{exo} [°C]	T_m [°C]	ΔH_f [J g^{-1}]	T_g [°C]	T_m [°C]	E' [MPa]
**S1**	-6.3	-	169.4	20.8	22.9	165.2	22.73
**S2**	-11.2	-	176.8	12.0	15.7	167.8	10.0
**S3**	-12.3	36.9	167.9	10.7	12.0	158.0	6.5
**S4**	-14.3	35.1	166.8	9.0	11.7	156.9	3.2
**S5**	-14.7	36.2	164.6	6.9	3.5	137.5	2.8

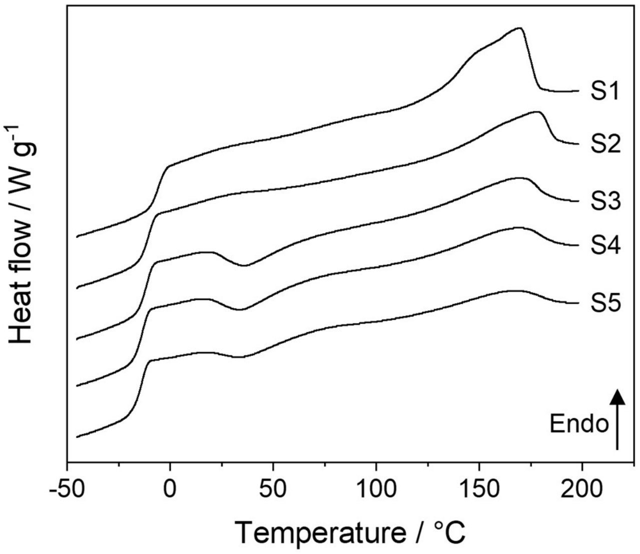

Figure S3. DSC thermographs of the PTUs **S1**-**S5**. The second heating run is shown.

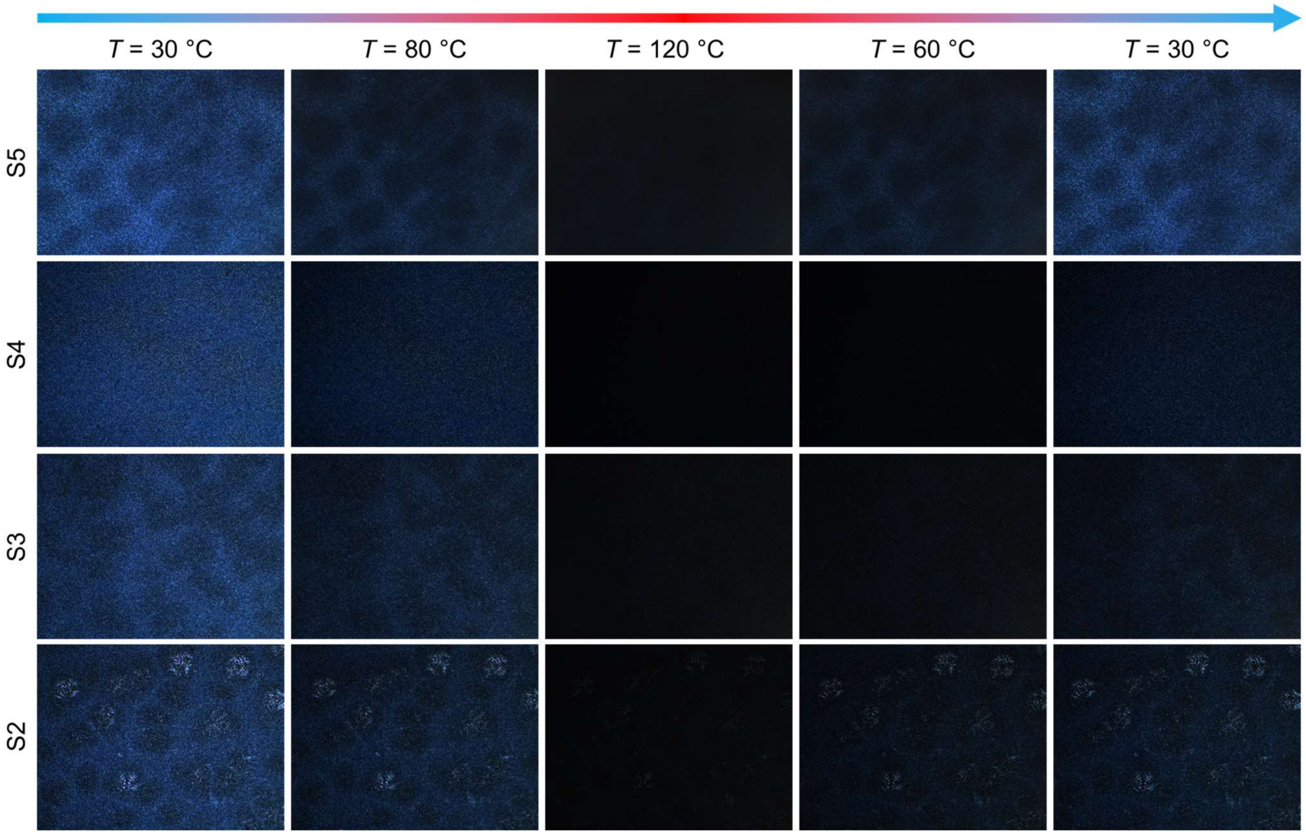

Figure S4. POM images of PTUs **S2**-**S5**. Upon heating, the birefringence disappears over a wide temperature range (60 to 120 °C) that reappears upon cooling, although showing thermal hysteresis. Poor solubility of PTU **S1** prevented sample fabrication.

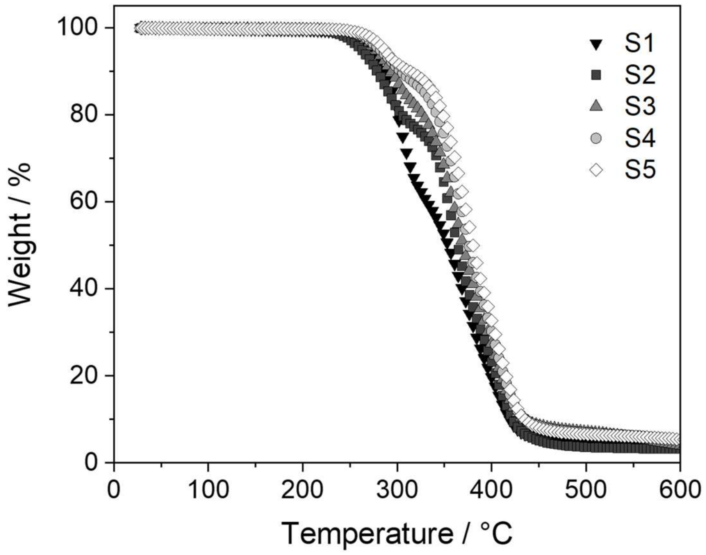

Figure S5. TGA profiles of PTUs **S1**-**S5**.

Table S6. Summary of mechanical properties of the thermoplastic PTUs **S1**-**S5**.

PTU	Young's modulus [MPa]	Tensile strength [MPa]	Elongation at break
**S1**	67.4	44.8	4.3
**S2**	25.1	24.5	5.2
**S3**	14.7	21.0	5.6
**S4**	6.7	13.5	5.9
**S5**	3.9	9.3	9.7

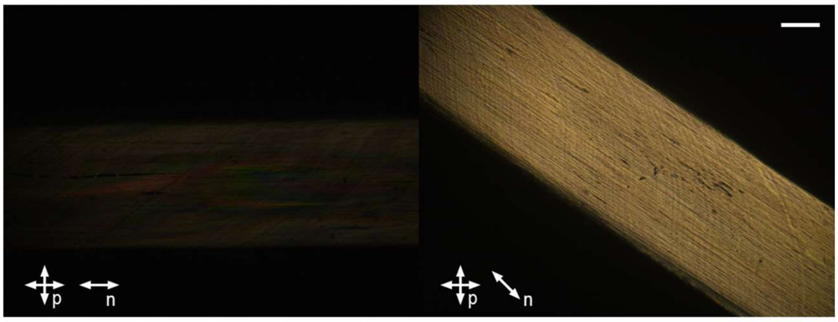

Figure S6. POM images of programmed PTU **S5** under cross polarizers. The crossed arrows indicate the polarizer directions (**p**), and the single arrows the molecular director of the aligned material (**n**). The scale bar is 200 µm.

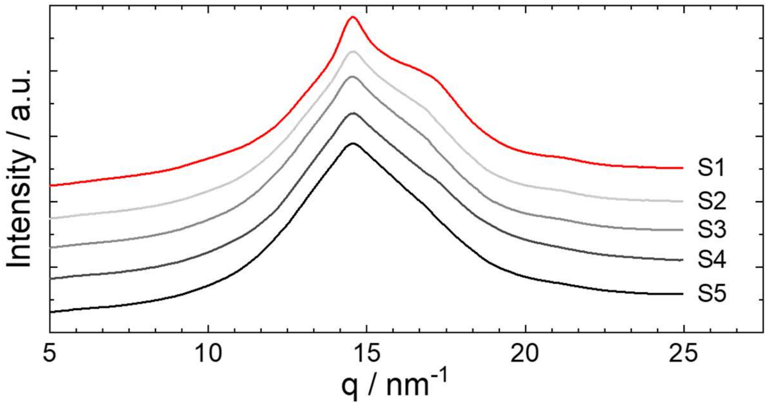

Figure S7. 1D WAXS diffractograms of polydomain PTUs **S1**-**S5**.

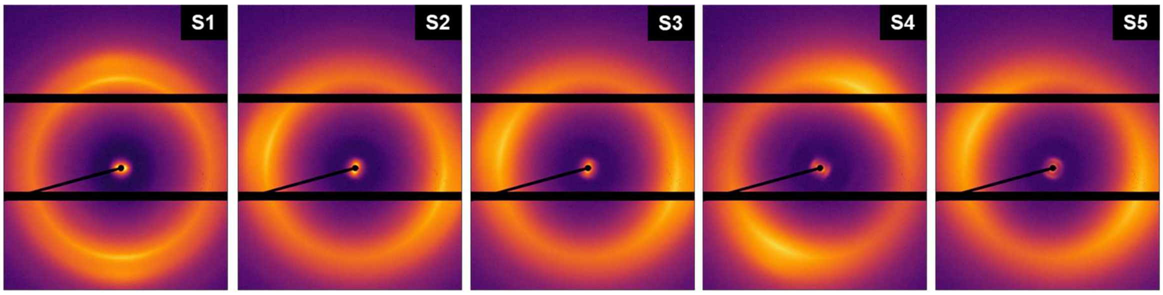

Figure S8. 2D WAXS diffractograms of polydomain PTUs **S1**-**S5**. The observed orientation is attributed to the sample preparation and handling.

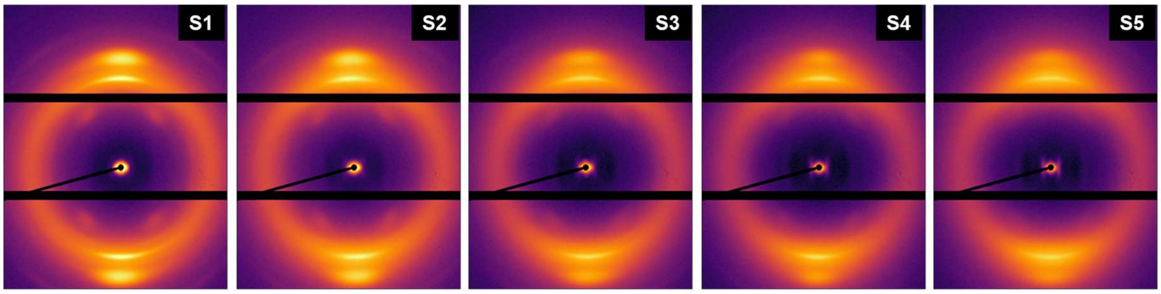

Figure S9. 2D WAXS diffractograms of programmed PTUs **S1**-**S5**. The molecular director of the aligned materials is horizontal.

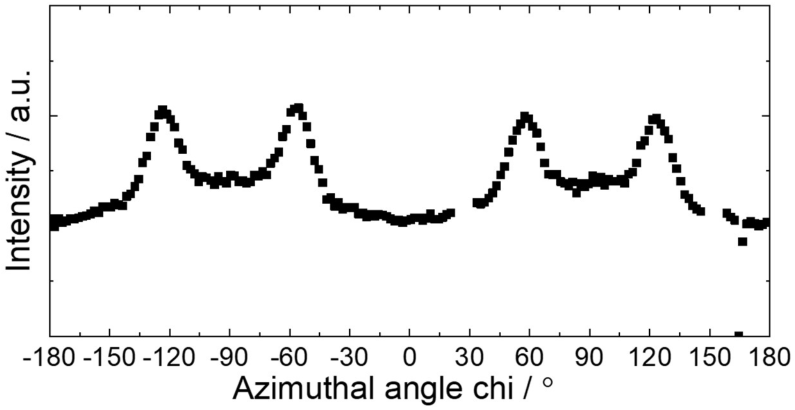

Figure S10. Azimuthal profile of the 2D WAXS diffractogram at q = 10.0-10.5 nm^{-1} for PTU **S1.**

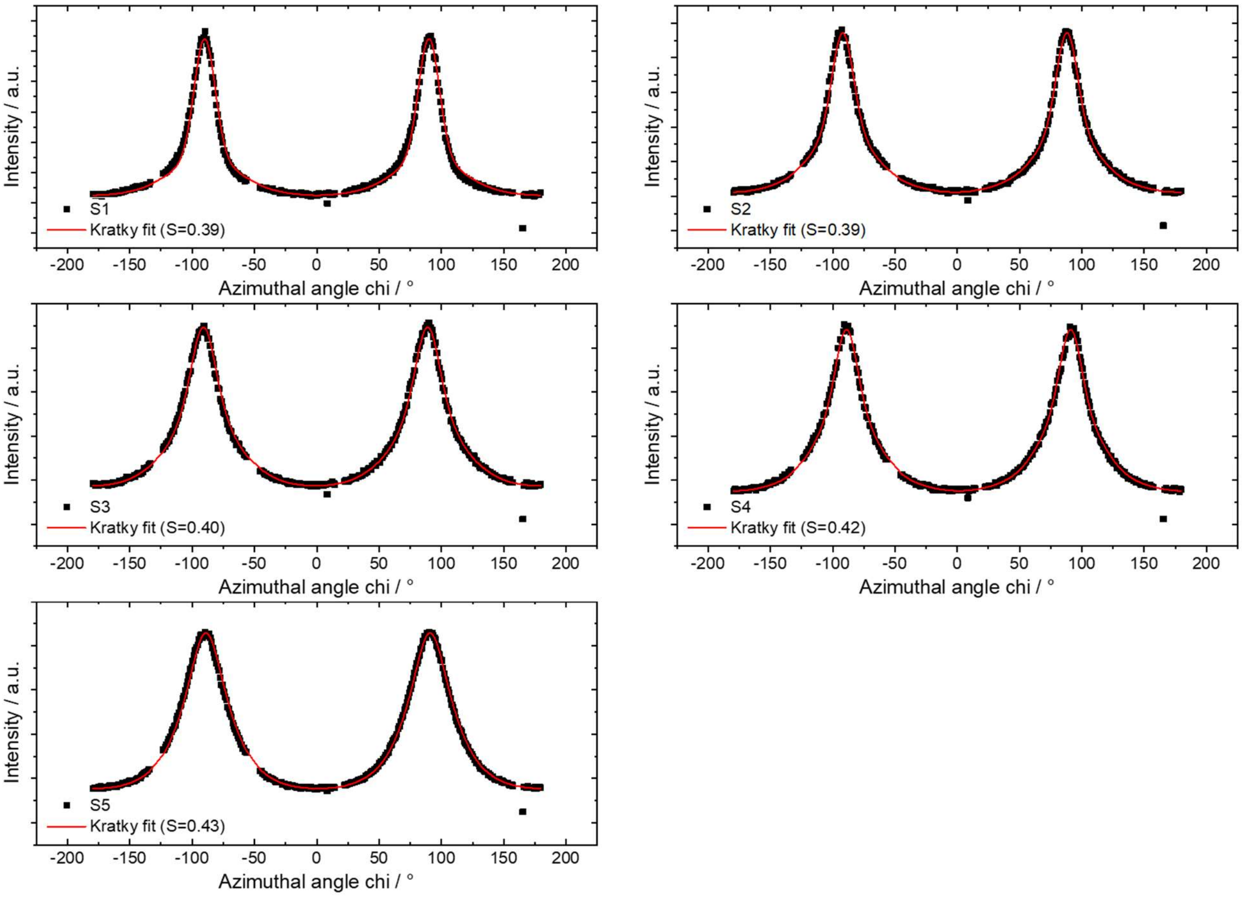

Figure S11. Azimuthal profiles of the 2D WAXS patterns at q = 14.3-14.6 nm^{-1} for PTUs **S1**-**S5**. The order parameter was obtained by fitting a Kratky function over the azimuthal angle by a custom-made script.

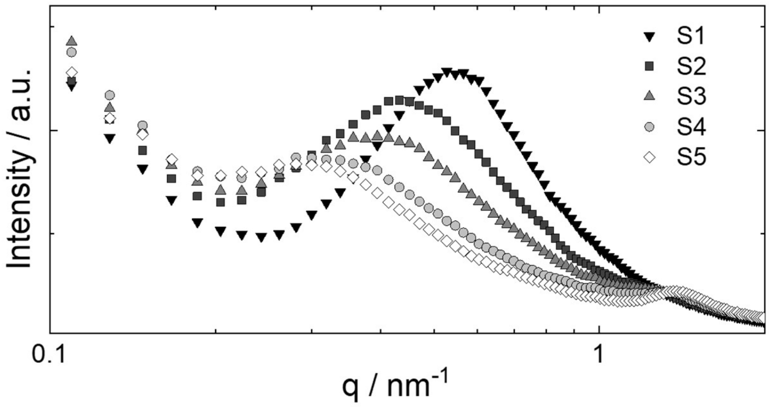

Figure S12. 1D MAXS diffractograms of polydomain PTUs **S1**-**S5**. An additional peak is observed at q = 1.37 nm^{-1} originating from the spacing between the mesogens within the soft segment (d = 4.6 nm). For samples with larger LC segment lengths, the signal is stronger.

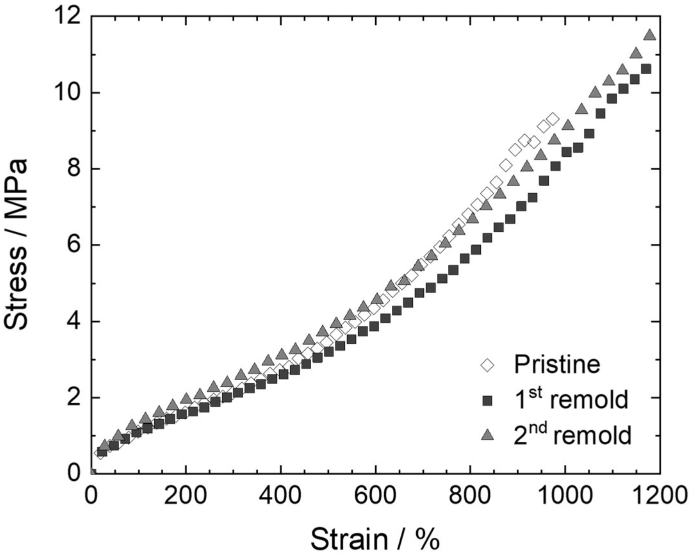

Figure S13. Stress-strain curves at room temperature of pristine and remolded samples.

Supplementary Video Captions

Video S1. Cyclic thermal actuation of thermoplastic PTU **S5** while lifting 5g.

Video S2. Thermal actuation of thermoplastic PTU **S5** while lifting various weights of 5g, 10g, 15g, 20g, 25g, and 30g.

Video S3. Thermal actuation of the reprogrammed twisted actuator.

Video S4. Thermal actuation of the reprogrammed patterned actuator.

Video S5. Thermal actuation of the reprogrammed and welded twisted actuator.

References

[1] M. T. Sims, L. C. Abbott, R. M. Richardson, J. W. Goodby, J. N. Moore, *Liq. Cryst.* **2019**, *46*, 11–24.

[2] M. O. Saed, R. H. Volpe, N. A. Traugutt, R. Visvanathan, N. A. Clark, C. M. Yakacki, *Soft Matter* **2017**, *13*, 7537–7547.

[3] Z. Liu, L. He, G. Guo, Q. Gui, Y. Yuan, H. Zhang, *Polymer* **2021**, *215*, 123420.

[4] B. Ni, H. Lou Xie, J. Tang, H. L. Zhang, E. Q. Chen, *Chem. Commun.* **2016**, *52*, 10257–10260.

[5] C. Chen, Y. Liu, X. He, H. Li, Y. Chen, Y. Wei, Y. Zhao, Y. Ma, Z. Chen, X. Zheng, H. Liu, *Chem. Mater.* **2021**, *33*, 987–997.

[6] W. Shen, J. Liu, B. Du, H. Zhuo, S. Chen, *J. Mater. Chem. A* **2021**, *9*, 15087–15094.

[7] L. Wang, Y. Zhou, S. Ma, H. Zhang, *J. Mater. Chem. C* **2021**, *9*, 13255–13265.

[8] M. Pozo, L. Liu, M. Pilz da Cunha, D. J. Broer, A. P. H. J. Schenning, *Adv. Funct. Mater.* **2020**, *30*, 2005560.

Author Contributions

S. J. D. L performed the experimental work and wrote the manuscript. D. J. M. and A. P. H. J. S. supervised the work and revised the final manuscript.
